# Association of anaemia with indoor air pollution among older Indian adult population: multilevel modelling analysis of nationally representative cross-sectional study

**DOI:** 10.1186/s12877-024-05171-2

**Published:** 2024-06-29

**Authors:** Pritam Halder, Madhur Verma, Saumyarup Pal, Amit Kumar Mishra, Trideep Jyoti Deori, Riya Biswas, Jaya Tiwari, Anshul Mamgai, Shivani Rathor, Manish Chandra Prabhakar

**Affiliations:** 1 Department of Community Medicine and School of Public Health, Postgraduate Institute of Medical Education and Research, Chandigarh, 160012 India; 2 Department of Community and Family Medicine, All India Institute of Medical Sciences, Bathinda, Punjab 151001 India; 3 Department of Geriatric Medicine, All India Institute of Medical Sciences, New Delhi, 110049 India; 4 School of Environmental Sciences, Jawaharlal Nehru University (JNU), New Delhi, 110067 India; 5 Ministry of Health & Family Welfare, Regional Office for Health & Family Welfare, Six Mile, Guwahati, 781037 India; 6 Centre for Community Medicine, All India Institute of Medical Sciences, New Delhi, 110049 India; 7 Department of Medical Parasitology, Postgraduate Institute of Medical Education and Research, Chandigarh, 160012 India

**Keywords:** Anaemia, Air pollution, Indoor air pollution, IAG, Modelling, LASI, Propensity score matching, Nested multilevel

## Abstract

**Introduction:**

Anaemia is a disease of public health importance with multi-causal pathways. Previous literature suggests the role of indoor air pollution (IAP) on haemoglobin levels, but this has been studied less due to logistic constraints. A high proportion of the population in developing countries, including India, still depends on unclean fuel, which exacerbates IAP. The objective was to study the association between anaemia and IAP among the older Indian adult population (≥ 45 years) as per gender.

**Methods:**

Our study analysed the nationally representative dataset of the Longitudinal Ageing Study in India (LASI 2017–18, Wave-1). We have documented the association of anaemia (outcome variable) with IAP (explanatory variable). To reduce the confounding effects of demographic and socioeconomic; health related and behavioural covariates; propensity score matching (PSM) was conducted. Nested multilevel regression modelling was conducted. States and union territories were categorised cross tabulated as low, middle and high as per anaemia and IAP exposure. P value < 0.05 was considered statistically significant. SATA version 17 was used for analysis.

**Results:**

More than half (52.52%) of the participants were exposed to IAP (male (53.55%) > female (51.63%)). The odds of having anaemia was significantly 1.19 times higher (AOR 1.19 (1.09–1.31)) among participants using unclean/ solid fuel. The adjusted odds were significantly higher among participants exposed to pollution-generating sources (AOR 1.30; 1.18–1.43), and household indoor smoking (AOR 1.17 (1.07–1.29). The odds of having anaemia were significantly higher (AOR 1.26; 1.15–1.38) among participants exposed to IAP, which was higher in males (AOR 1.36; 1.15–1.61) than females (AOR 1.21; 1.08–1.35). Empowered Action Group (EAG) states like Uttar Pradesh, Chhattisgarh, Madhya Pradesh, Bihar had both high anaemia and IAP exposure.

**Conclusion:**

This study established the positive association of anaemia with indoor air pollution among older Indian adults through a nationally representative large dataset. The association was higher among men. Further research is recommended to understand detailed causation and to establish temporality. It is a high time to implement positive intervention nationally to decrease solid/ unclean fuel usage, vulnerable ventilation, indoor smoking, IAP and health hazards associated with these with more focused actions towards EAG states.

**Supplementary Information:**

The online version contains supplementary material available at 10.1186/s12877-024-05171-2.

## Introduction

Anaemia is essentially a homeostatic imbalance in the hemoglobin concentration (< 12 g/dL in women and 13 g/dL in men) whereby the production of erythrocytes is outpaced by destruction or loss of erythrocytes. It leads to poor health, economic loss and social burden [1]. It is the result of a wide variety of causes that can be isolated, but often they coexist. Globally, the most common cause of nutrient deficiency anaemia is due to iron deficiency, although other conditions, such as folate, vitamin B12 and vitamin A deficiencies, chronic inflammation, parasitic infections, and inherited disorders can cause anaemia [2].

Prevalence of anaemia is higher in developing countries, south Asian countries contributing 37.5% of global anaemia [3]. Poverty, inadequate diet, diseases, pregnancy/lactation and poor access to health services are some of the key factors contributing to the high burden [4]. Globally prevalence of anaemia was 12–17% among older adults (≥ 65 years); 40% hospitalised and 47% patients in nursing home had anaemia [5]. Given that the world's population is getting older due to demographic shifts, the overall burden of disease related to anaemia among the elderly is probably only going to increase [6].

The emergence and advancement of anaemia may be influenced by common environmental exposures, such as air pollution. Numerous extremely common chronic diseases, such as respiratory, mental, and cardiovascular conditions, are recognised to be at risk due to ambient air pollution [7]. In developing nations, ambient air pollution has had a severe negative influence on public health [8]. Industrialisation in these areas, propelled by economic expansion, has resulted in significant rises in air pollution levels, frequently an order of magnitude more than those found in industrialised nations. The latter has exacerbated bad health outcomes and increased the risk to human life [9]. There are few studies on the relationship between air pollution and anaemia, and the majority of the earlier studies concentrated on the population of children or short-term exposures [10–12]. There were very few study showing among older adults (≥ 45 years) [13, 14]. Hence, we have conducted this study to show the association of anaemia with indoor air pollution among older Indian adults (≥ 45 years).

## Objective

To determine the association with anaemia with Indoor air pollution

- among older Indian population (≥ 45 years).
- among male and female older Indian population (≥ 45 years).

## Methodology

Data source: LASI-1st wave is a longitudinal survey with a national representation that intends to collect detailed information on the psychological, social, economic, and health aspects of ageing in India from all the states and union territories. It was developed to fill the information vacuum regarding thorough and internationally comparable survey data on India's ageing population. The study, which is the biggest of its kind in the world and the first of its kind in India, evaluates the scientific evidence in the context of variables like demographics, household economic status, chronic health conditions, symptom-based health conditions, functional health, mental health (cognition and depression), biomarkers, healthcare utilisation, family and social networks, social welfare programmes, employment, retirement, satisfaction, and life expectations. The survey intends to follow a representative sample of the older adult population every two years for the following 25 years, with a revised sample size to account for attrition due to death, migration, non-reachable, and non-response [15]. The funding agencies were National Institute on Ageing, the Government of India's Ministry of Health and Family Welfare, and the United Nations Population Fund. The University of Southern California, the International Institute for Population Sciences, and the Harvard T.H. Chan School of Public Health were the contributors.

### Study population

**Fig. 1:**
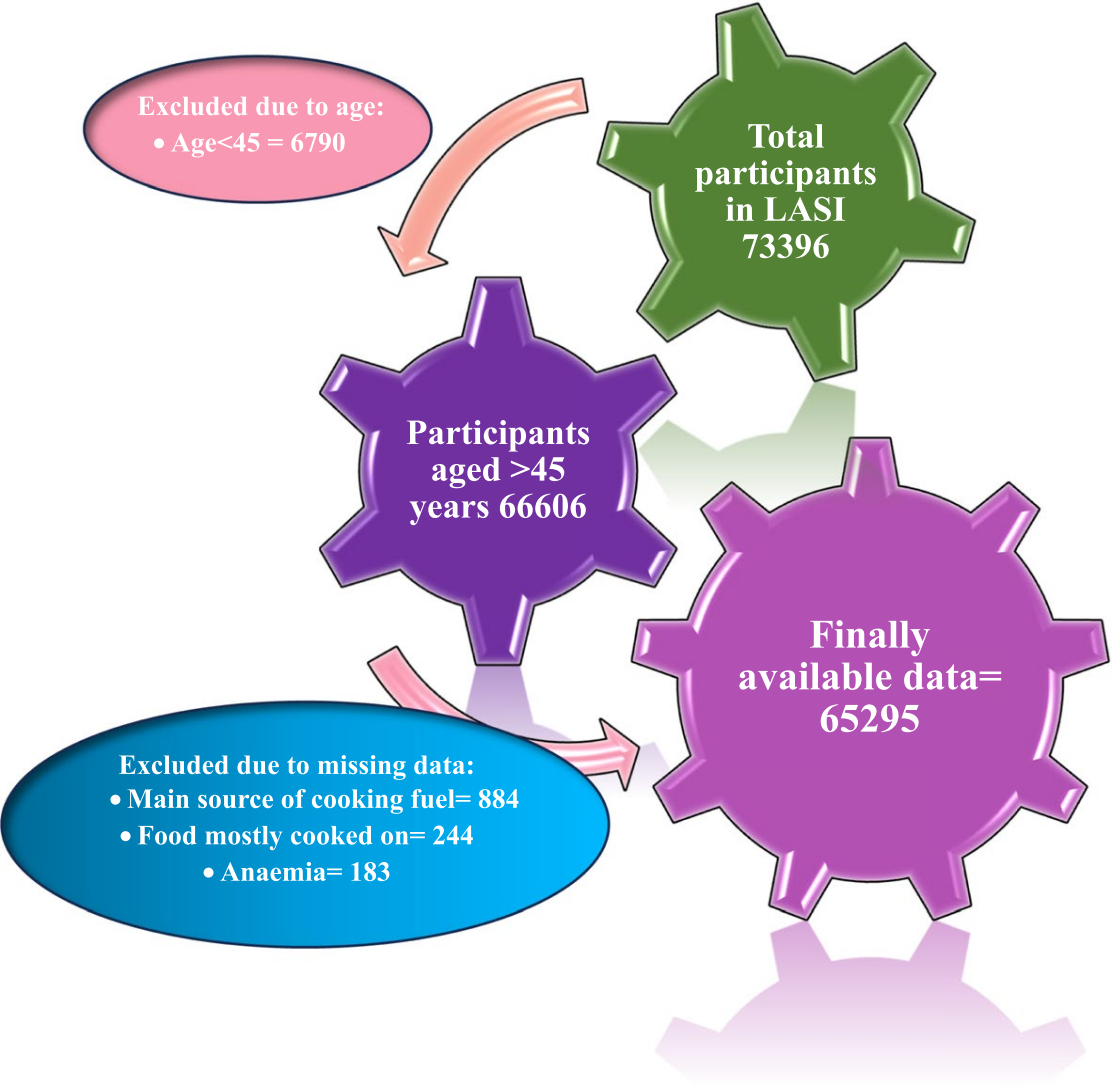
Flowchart showing participants selection process in this study

A total of 73,396 adult Indians were surveyed from during April 2017-December 2018 (Sikkim: 2020–2021). Out of them, 65,295 participants were included for the present study. Details of study flow and sample selection with missing data handling (row wise complete deletion/ complete case analysis) were documented in Fig. 1.

### Study variables

#### Outcome variable

The outcome variable of choice was anaemia. Self-reported anaemia prevalence was obtained by questioning ‘In the past 2 years, have you had anaemia?’ Answering ‘yes’ was considered as anaemia present.

#### Explanatory variables

Participants exposed to indoor air pollution (IAP) was the explanatory variable of choice. IAP includes contamination of the air from physical, chemical, and biological sources. A distinct component on IAP was surveyed as part of the LASI study. Six questions from the LASI survey were used to calculate IAP. There were two questions concerning the fuel utilised for cooking and other purposes:

(i)” What is your main source of cooking fuel?” and.

(ii) “What are those other sources of fuel used for other purposes (such as boiling water for bathing, lighting, etc.)?” (Responses: Liquefied Petroleum Gas (LPG), Biogas, Kerosene, Electric, Charcoal/Lignite/Coal, Crop residue, Wood/Shrub, Dung cake, do not cook at home, Other, please specify). ‘Fuel type’ was generated considering LPG, Biogas, and Electric methods as clean fuels and the rest as unclean or solid fuels. ‘Pollution generating source’ was generated from type of oven used: (iii) “In this household, is food mostly cooked on a mechanical stove, on a traditional chullah or over an open fire?” (Responses: Mechanical Stove/Improved cook stove, Traditional chullah, Open fire, Other, please specify). Traditional chullah and opened fire was taken as the higher pollution generating source. Next two questions were about place of cooking and ventilation: (iv) “Is the cooking usually done in the house, in a separate building, or outdoors?” (Responses: In the house, In a separate building, Outdoors, Other, please specify); (v) “Is the cooking mainly done under a traditional chimney, exhaust fan, electric chimney or near window/door?” (Responses: Traditional chimney, Electric chimney, Exhaust fan, Near window/door, None). No ventilation with in-house cooking was considered as vulnerable ventilation. Next question was on ‘Household Indoor Smoking’: (vi) “Does any usual member of your household smoke inside the home?”(Responses: Yes, No). Thus, all six factors were used to generate ‘Indoor Air Pollution’: exposed (Participants using unclean/ solid fuel for cooking and others by utilising traditional chullah or open fire and inhouse cooking without any ventilation system along with presence of indoor smoking.) and non-exposed participants. Thus ‘fuel type’, ‘pollution generating source’, ‘vulnerable ventilation’, ‘household indoor smoking’ and’indoor air pollution’ were considered as explanatory variables.

#### Covariates

These variables were categorised into demographic and socioeconomic, health related and behavioural factors. Under demographic and socioeconomic factors, we have included- age group (45–64, ≥ 65 years), gender (male, female), education (illiterate, less than primary. primary completed, middle completed, secondary school, higher secondary, and Diploma/ graduate), residence (rural, urban), marital status (unmarried, married/ in live-in, Widow/ separated/ divorced), MPCE (monthly per capita expenditure- poorest, poorer, middle, richer, richest) quintile, health insurance (no, yes), occupation (unemployed, professional and semi-professional- ‘legislators and senior officials, professionals, technicians and associate professionals’, clerical and skilled- ‘clerks, service workers and shopkeepers, skilled agriculture and fishery workers, craft and related trade worker, plant and machine operator’, unskilled). Under health-related factors, we have included- physical activity (everyday, once per week, 1–3 times per week, once per month, never), self-rated health (excellent, very good, good, fair, poor) and multimorbidity. Following chronic morbidities were included- hypertension, diabetes, cancer, chronic lung diseases (e.g.- chronic obstructive pulmonary disease, asthma, chronic bronchitis, other chronic lung problems), chronic heart disease (e.g.- congestive heart failure, myocardial infarction, heart attack, other chronic heart diseases), stroke, musculoskeletal disorder (MSD e.g.- rheumatism, arthritis, osteoporosis, other chronic joint or bone disorders), dyslipidaemia (high cholesterol), thyroid disorders, Chronic renal failure, visual impairment and hearing impairment. Interviewer asked related question about chronic health conditions/ morbidities with dichotomous answers (no/ yes)- “Has any health professional ever diagnosed you with the following chronic conditions or diseases?” Participants having at least two chronic health conditions were described as multimorbidity. Under behavioural factors, we have included- tobacco abuse (no, yes) and alcohol abuse (no, yes).

#### Statistical analysis

Data was analysed in Stata version 17 (StataCorp. 2017. Stata Statistical Software: Release 17. College Station, TX: StataCorp LP.). The characteristics of the participants were described as mean (standard deviation) for continuous variable frequencies and percentages for categorical variables. Individual sample weights were considered during the analysis. Chi-square *p*-value was estimated. Indian states and union territories were categorised into low (0 to 33rd percentile), middle (34 to 66th percentile) and high (67 to 100th percentile) as per anaemia and indoor air pollution each. We have produced Indian map to document these categories with Microsoft excel. We have further cross tabulated them to obtain common categories.

To reduce the confounding effects of covariates, we have used propensity score matching (PSM). We have used nested multilevel modelling to show the association between anaemia and IAP. We have used total 4 models. In the Model-1, we have included IAP. In the Model-2, Model-3 and Model-4; we have subsequently added demographic and socioeconomic, health related and behavioural factors, respectively after assessing the multicollinearity among explanatory variables using the VIF (Variance inflation factor), and variables > 5 indicate a high correlation and were omitted. (Self-related health and marital status had VIF > 5. (Supplementary Table S1)) Hence, all the covariates except these two were included in the final association. We have also documented pseudo R2, log-likelihood, likelihood ratio, AIC (Akaike Information Criterion) and BIC (Bayesian Information Criterion) to evaluate the best model. We have then documented the association as per gender. We reported state wise variations of this association. *P*-value < 0.05 were considered as statistically significant.

#### Ethical statement

Dataset is freely available in the public domain, ethical approval for the present study was not deemed necessary. However, the ethical approval to conduct LASI was given by the Indian Council of Medical Research's (ICMR) Central Ethics Committee on Human Research (CECHR) [15].

## Results

The mean (SD) age of the participants were 60.27 (10.77) years. Around 50.82% of participants were illiterate, which was higher in females (65.32%). Around 69.75% of participants resided in the rural area. Almost three-fourth of the participants were married. Only 2.31% of participants had health insurance. Almost half of the participants (49.90%) were unemployed, which was higher among females (67.11%). Almost one-fourth (25.07%) of the participants exercised every day, which was higher among males (32.92%). Multimorbidity was 36.55% overall, higher among females (38.42%). Around 37.26% and 15.00% of participants had a history of tobacco abuse and alcohol abuse, respectively, which was more in men in both cases. (Table 1).

**Table 1:** Socio-demographic characteristics of the adults aged ≥ 45 years included in the Longitudinal Aging Study in India (2017–18)

Variable	Overall	Male	Female	Chi-square *p*-value
	***N*(%)**	***N*(%)**	***N*(%)**	
**Total participants**^**b**^	65,295 (100.00)	30,452 (46.64)	34,843 (53.36)	-
**Age (years)**^**a**^	60.27 (10.77)	60.65 (10.72)	59.94 (10.79)	-
**Age group (years)**^**b**^				
45–59	42,498 (65.09)	19,394 (63.69)	23,096 (66.28)	< 0.001
>60	22,797 (34.91)	11,058 (36.31)	11,747 (33.72)	
**Education**^**b**^				
Illiterate	33,184 (50.82)	10,326 (33.91)	22,761 (65.32)	< 0.001
Less than primary	7185 (11.00)	4083 (13.41)	3115 (8.94)	
Primary completed	7937 (12.16)	4543 (14.92)	3410 (9.79)	
Middle completed	5358 (8.21)	3465 (11.38)	1911 (5.49)	
Secondary school	5075 (7.77)	3418 (11.22)	1677 (4.81)	
Higher secondary	2848 (4.36)	2005 (6.58)	855 (2.45)	
Diploma/ Graduate	3708 (5.68)	2612 (8.58)	1114 (3.19)	
**Residence**^**b**^				
Rural	45,546 (69.75)	21,501 (70.61)	24,050 (69.02)	0.008
Urban	19,749 (30.25)	8951 (29.39)	10,793 (30.98)	
**Marital Status**^**b**^				
Unmarried	788 (1.21)	425 (1.40)	364 (1.04)	< 0.001
Married/ in live -in	48,341 (74.03)	26,679 (87.61)	21,740 (62.39)	
Widow/ separated/ divorced	16,166 (24.76)	3348 (10.99)	12,739 (36.56)	
**MPCE quintile**^**b**^				
Poorest	13,623 (20.86)	6168 (20.25)	7421 (21.39)	0.147
Poorer	13,949 (21.36)	6480 (21.28)	7467 (21.43)	
Middle	13,211 (20.23)	6124 (20.11)	7087 (20.34)	
Richer	12,655 (19.38)	5974 (19.62)	6683 (19.18)	
Richest	11,857 (18.16)	5706 (18.73)	6155 (17.67)	
**Health insurance**^**b**^				
No	63,787 (97.69)	29,241 (96.02)	34,536 (99.12)	< 0.001
Yes	1508 (2.31)	1211 (3.98)	307 (0.88)	
**Occupation**^**b**^				
Unemployed	32,585 (49.90)	9088 (29.84)	23,381 (67.11)	< 0.001
Professional and semi-professional	1755 (2.69)	1352 (4.44)	413 (1.18)	
Clerical and skilled	18,764 (28.74)	12,373 (40.63)	6460 (18.54)	
Unskilled	12,191(18.67)	7639 (25.08)	4589 (13.17)	
**Physical activity**^**b**^				
Everyday	16,367 (25.07)	10,024 (32.92)	6388 (18.33)	< 0.001
More than once / week	4345 (6.65)	2539 (8.34)	1816 (5.21)	
Once / week	2387 (3.65)	1390 (4.56)	1002 (2.88)	
1–3 times /month	3575 (5.47)	1831(6.01)	1747 (5.01)	
Never	38,622 (59.15)	14,669 (48.17)	23,890 (68.56)	
**Self-rated health**^**b**^				
Excellent	2766 (4.29)	1589 (5.29)	1183 (3.44)	< 0.001
Very good	11,354 (17.63)	5681(19.92)	5681 (16.52)	
Good	24,139 (37.47)	11,416 (38.02)	12,726 (37.01)	
Fair	19,035 (29.55)	8279 (27.57)	10,745 (31.25)	
Poor	7121 (11.05)	3063 (10.20)	4053 (11.79)	
**Multimorbidity**^**b**^				
No	41,431 (63.45)	19,989 (65.64)	21,455 (61.58)	< 0.001
Yes	23,864 (36.55)	10,463 (34.36)	13,388 (38.42)	
**Tobacco abuse**^**b**^				
No	40,965 (62.74)	12,813 (42.08)	28,032 (80.45)	< 0.001
Yes	24,330 (37.26)	17,639 (57.92)	6811 (19.55)	
**Alcohol abuse**^**b**^				
No	55,504 (85.00)	21,502 (70.61)	33,919 (97.35)	< 0.001
Yes	9791 (15.00)	8950 (29.39)	924 (2.65)	

a = mean (SD)

b = *N* (%)

Unclean/ solid fuel usage was 38.35% overall. Higher vulnerable ventilation was seen among 18.09% participants. Around 48.90% and 25.00% participants were exposed to pollution generating source and household indoor smoking, respectively. More than half (52.52%) of the participants were exposed to the indoor air pollution (IAP). All the above were higher among males except in using unclean/ solid fuel and higher vulnerable ventilation. (Table 2).

**Table 2:** Distribution of Indian population as per indoor air pollution

Characteristics		Overall *N* = 65,295	Male *N* = 30,452	Female *N* = 34,843
		*N* (%)	*N* (%)	*N* (%)
Fuel type	Clean	40,251 (61.65)	18,933 (62.17)	21,322 (61.19)
	Unclean/ Solid	25,044 (38.35)	11,519 (37.83)	15,521 (38.81)
Vulnerable ventilation	Lower	53,486 (81.91)	24,979 (82.03)	28,508 (81.82)
	Higher	11,809 (18.09)	5473 (17.97)	6335 (18.18)
Pollution generating source	No	33,368 (51.10)	15,542 (51.04)	17,826 (51.16)
	Yes	31,927 (48.90)	14,910 (48.96)	17,017 (48.84)
Household* Indoor Smoking	No	48,969 (75.00)	22,013 (72.29)	26,940 (77.32)
	Yes	16,326 (25.00)	8439 (27.71)	7903 (22.68)
Indoor Air Pollution*	No	31,005 (47.48)	14,144 (46.45)	16,855 (48.37)
	Yes	34,290 (52.52)	16,308 (53.55)	17,988 (51.63)

^*^Chi-square *p*-value < 0.05 = significant

**Fig. 2:**
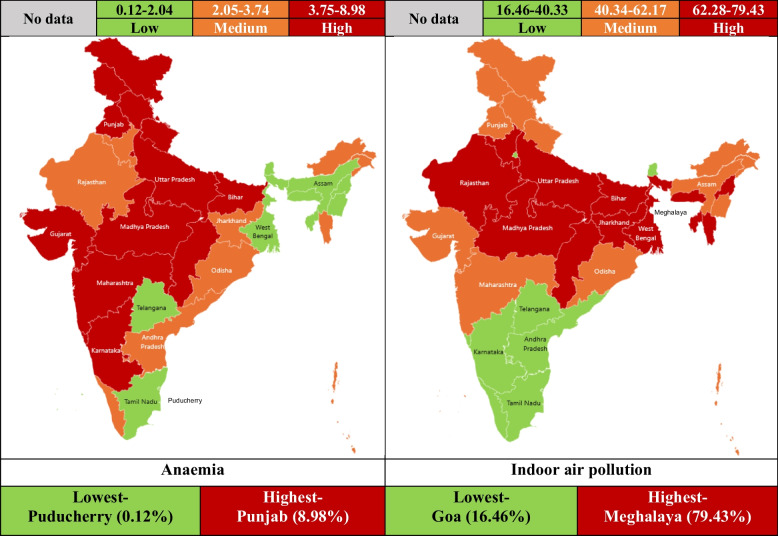
State/ Union Territory wise distribution of participants as per anaemia and indoor air pollution

IAP was highest in Meghalaya (79.43%) and lowest in Goa (16.46%). Self-reported anaemia was present among 2962 (4.54%) participants. Anaemia was more prevalent among female (5.75%) than male (3.12%) participants. The anaemia prevalence was highest in Punjab (8.98%) and lowest in Puducherry (0.12%) (Supplementary Table S2). Figure 2 represents the categorisation of Indian states and union territories as per anaemia and IAP. Central states like Uttar Pradesh, Chhattisgarh, Madhya Pradesh, Bihar had both high anaemia and IAP. In the contrary both low anaemia and IAP was documented in Puducherry, Sikkim, Telangana, Daman and Diu, Tamil Nadu. Though the IAP was high in Nagaland, Meghalaya, Tripura, West Bengal; still the anaemia prevalence was low. Though the LAP was low in Goa, Delhi, Karnataka; anaemia was high. (Table 3).

**Table 3:** Categorisation of states and union territories by anaemia and indoor air pollution

Category		Indoor air pollution		
		Low	Medium	High
Anaemia	Low	Puducherry, Sikkim, Telangana, Daman and Diu, Tamil Nadu	Lakshadweep, Manipur, Assam	Nagaland, Meghalaya, Tripura, West Bengal
	Medium	Andhra Pradesh, Arunachal Pradesh, Kerala, Chandigarh	Odisha, Andaman and Nicobar Islands, Dadra and Nagar Haveli	Jharkhand, Mizoram, Haryana, Rajasthan
	High	Goa, Delhi, Karnataka	Uttarakhand, Maharashtra, Jammu and Kashmir, Himachal Pradesh, Gujarat, Punjab	Uttar Pradesh, Chhattisgarh, Madhya Pradesh, Bihar

The coefficient for the estimated treatment effect of IAP over anaemia across population (ATE) is 0.0083. After adjusting for covariates, individuals exposed to indoor air pollution (IAP) have significantly increased risk of anaemia compared to non-exposed individuals. The estimated effect size is approximately 0.83% higher odds of having anaemia in the participants exposed to IAP. (Table 4).

**Table 4:** Propensity Score matching (PSM) between anemia (outcome variable) and indoor air pollution (treatment/ explanatory variable) adjusted with covariates

Anemia	Coefficient	95% Confidence Interval	*p*-value
ATE: Indoor Air pollution	**0.0083**	**0.0043–0.0123**	** < 0.001**

**Fig. 3:**
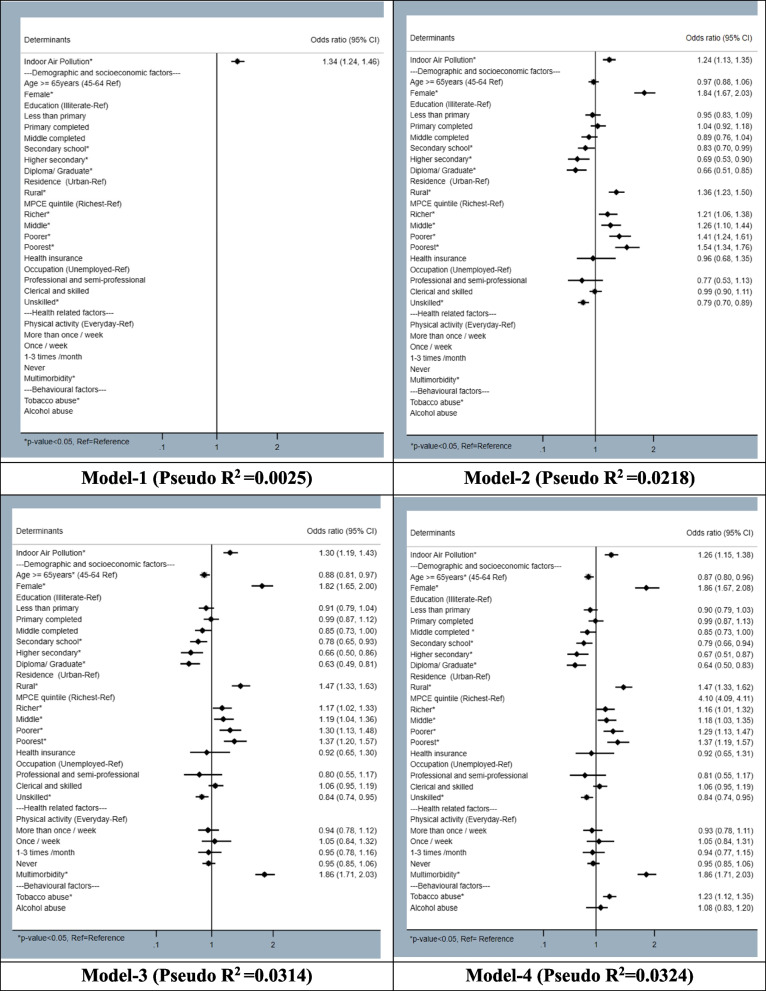
Nested multilevel modelling showing association of anaemia with indoor air pollution and various determinants among Indian population aged ≥ 45 years

We have reported the association of anaemia and IAP using nested multilevel modelling using 4 models. Out of all, Model-4 was the best with highest pseudo R^2^ and log-likelihood; and lowest AIC (Akaike Information Criterion) and BIC (Bayesian Information Criterion) values with statistically significant p-value. The odds of having anaemia was significantly 1.19 times higher (AOR 1.19 (1.09–1.31)) among participants using unclean/ solid fuel than clean fuel; which was higher among male (AOR 1.42 (1.20–1.67)). The odds of having anaemia was significantly higher (AOR 1.30 (1.18–1.43)) among participants exposed to pollution generating source, which was higher in male (1.58 (1.33–1.88)). The odds was significantly higher (AOR 1.17 (1.07–1.29)) among participants exposed to household indoor smoking, which was higher in female (1.21 (1.08–1.35)). The odds of having anaemia was significantly 1.26 times higher (AOR 1.26 (1.15–1.38)) among participants exposed to indoor air pollution, which was higher in male (1.36 (1.15–1.61)) than female (1.21 (1.08–1.35)). (Fig. 3, Tables 5 and 6).

**Table 5:** Nested multilevel modelling showing association of anaemia with various components of indoor air pollution and various determinants among Indian population aged ≥ 45 years

**Characteristics**		**Anaemic**			
		**Model-1**	**Model-2**	**Model-3**	**Model-4**
		**Odds ratio (95% CI)**	**Odds ratio ** **(95% CI))**	**Odds ratio ** **(95% CI)**	**Odds ratio ** **(95% CI)**
**Overall (>45 years)**^**a**^					
Fuel type	Clean	Reference	Reference	Reference	Reference
	Unclean/ Solid	1.27 (1.16-1.38)*	.1.13 (1.03-1.24)*	1.21 (1.10-1.33)*	1.19 (1.09-1.31)*
	Pseudo R^2^	0.0015	0.0211	0.0305	0.0318
Vulnerable ventilation	Lower	Reference	Reference	Reference	Reference
	Higher	1.03 (0.93-1.15)	0.99 (0.89-1.09)	1.00 (0.90-1.12)	1.01 (0.90-1.12)
	Pseudo R^2^	0.0001	0.0208	0.940	0.905
Pollution generating source	No	Reference	Reference	Reference	Reference
	Yes	1.39 (1.29-1.51)*	1.24 (1.13-1.37)*	1.33 (1.21-1.46)*	1.30 (1.18-1.43)*
	Pseudo R^2^	0.0031	0.0218	0.0314	0.0326
Household Indoor Smoking	No	Reference	Reference	Reference	Reference
	Yes	1.23 (1.12-1.34)*	1.21 (1.10-1.32)*	1.23 (1.12-1.34)*	1.17 (1.07-1.29)*
	Pseudo R^2^	0.0010	0.0216	0.0307	0.0317
Indoor Air Pollution	Unexposed	Reference	Reference	Reference	Reference
	Exposed	1.34 (1.24-1.46)*	1.24 (1.13-1.35)*	1.30 (1.19-1.43)*	1.26 (1.15-1.38)*
	Pseudo R^2^	0.0025	0.0218	0.0314	0.0324
** Model**	**LL**	**LR**	***p*-value**	**AIC**	**BIC**
Model-1	-10700.75	52.63	<0.001	21405.51	21423.68
Model-2	-10492.84	415.83	<0.001	21023.68	21196.33
Model-3	-10390.71	204.26	<0.001	20829.42	21048.50
Model-4	-10380.01	21.39	<0.001	20812.03	21048.28

*CI* Confidence Interval, *LL* Log-likelihood, *LR* Likelihood Ratio, *AIC* Akaike Information Criterion and *BIC* Bayesian Information Criterion

Model 1- Association anaemia (outcome variable) with indoor air pollution/ IAP (explanatory variable)

Model 2- 1 + Demographic and socioeconomic factors (age group, gender, education, residence, mpce quintile, health insurance and occupation)

Model 3- Model 2 + Health related factors (physical activity and multimorbidity)

Model 4- Model 3 + Behavioural factors (tobacco and alcohol abuse)

*p-value<0.05= significant

**Table 6:** Nested multilevel modelling showing association of anaemia with various components of indoor air pollution and various determinants among Indian population aged ≥ 45 years as per gender

**Characteristics**		**Anaemic**				
		**Model-1**	**Model-2**	**Model-3**	**Model-4**	
		**Odds ratio (95% CI)**	**Odds ratio ** **(95% CI)**	**Odds ratio ** **(95% CI)**	**Odds ratio** ** (95% CI)**	
**Male**						
Fuel type	Clean	Reference	Reference	Reference	Reference	
	Unclean/ Soild	1.65 (1.43-1.90)*	1.37 (1.16-1.60)*	1.47 (1.25-1.73)*	1.42 (1.20-1.67)*	
	Pseudo R^2^	0.0063	0.0173	0.0266	0.0309	
Vulnerable ventilation	Lower	Reference	Reference	Reference	Reference	
	Higher	1.08 (0.89-1.29)	0.97 (0.80-1.17)	0.99 ((0.82-1.20)	0.99 (0.82-1.19)	
	Pseudo R^2^	0.001	0.0153	0.0237	0.0285	
Pollution generating source	No	Reference	Reference	Reference	Reference	
	Yes	1.84 (1.59-2.13)*	1.57 (1.32-1.86)*	1.66 (1.40-1.97)*	1.58 (1.33-1.88)*	
	Pseudo R^2^	0.0096	0.0191	0.0284	0.0323	
Household Indoor Smoking	No	Reference	Reference	Reference	Reference	
	Yes	1.28 (1.10-1.49)*	1.17 (1.01-1.37)*	1.21 (1.03-1.41)*	1.01 (0.85-1.19)	
	Pseudo R^2^	0.0014	0.0159	0.0244	0.0285	
Indoor Air Pollution	Unexposed	Reference	Reference	Reference	Reference	
	Exposed	1.71 (1.47-1.98)*	1.44 (1.22-1.69)*	1.52 (1.29-1.80)*	1.36 (1.15-1.61)*	
	Pseudo R^2^	0.0072	0.0180	0.0273	0.0303	
** Model**	**LL**	**LR**	***p*-value**	**AIC**	**BIC**	
Model-1	-3627.60	52.32	<0.001	7259.20	7275.85	
Model-2	-3587.83	79.55	<0.001	7211.65	7361.48	
Model-3	-3554.08	67.50	<0.001	7154.15	7345.60	
Model-4	-3543.19	21.78	<0.001	7136.37	7344.47	
**Female**						
Fuel type	Clean	Reference	Reference	Reference	Reference	
	Unclean/ Solid	1.11 (1.01-1.23)*	1.03 (0.92-1.15)	1.10 (0.98-1.23)	1.09 (0.98-1.23)	
	Pseudo R^2^	0.0003	0.0087	0.0185	0.0205	
Vulnerable ventilation	Lower	Reference	Reference	Reference	Reference	
	Higher	1.01 (0.89-1.15)	0.99 (0.87-1.13)	1.01 (0.89-1.15)	1.01 (0.89-1.16)	
	Pseudo R^2^	0.0001	0.0087	0.0183	0.0203	
Pollution generating source	No	Reference	Reference	Reference	Reference	
	Yes	1.23 (1.12-1.36)*	1.12 (1.01-1.25)*	1.20 (1.07-1.35)*	1.20 (1.07-1.34)*	
	Pseudo R^2^	0.0013	0.0090	0.0190	0.0210	
Household Indoor Smoking	No	Reference	Reference	Reference	Reference	
	Yes	1.28 (1.15-1.42)*	1.22 (1.09-1.36)*	1.23 (1.10-1.37*)	1.21 (1.08-1.35)*	
	Pseudo R^2^	0.0014	0.0096	0.0193	0.0211	
Indoor Air Pollution	Unexposed	Reference	Reference	Reference	Reference	
	Exposed	1.23 (1.12-1.36)*	1.16 (1.04-1.29)*	1.22 (1.09-1.36)*	1.21 (1.08-1.35)*	
	Pseudo R^2^	0.0013	0.0092	0.0192	0.0211	
** Model**	**LL**	**LR**	***p*-value**	**AIC**	**BIC**	
Model-1	-6930.82	18.28	<0.001	13865.64	13882.56	
Model-2	-6875.81	110.02	<0.001	13787.62	13939.88	
Model-3	-6806.44	138.75	<0.001	13658.87	13853.42	
Model-4	-6793.22	26.43	<0.001	13636.44	13847.91	

*CI* Confidence Interval, *LL* Log-likelihood, *LR* Likelihood Ratio, *AIC* Akaike Information Criterion and *BIC* Bayesian Information Criterion

Model 1- Association anaemia (outcome variable) with indoor air pollution/ IAP (explanatory variable)

Model 2- 1 + Demographic and socioeconomic factors (age group, education, residence, mpce quintile, health insurance and occupation)

Model 3- Model 2 + Health related factors (physical activity and multimorbidity)

Model 4- Model 3 + Behavioural factors (tobacco and alcohol abuse)

*p-value<0.05= significant

**Fig. 4:**
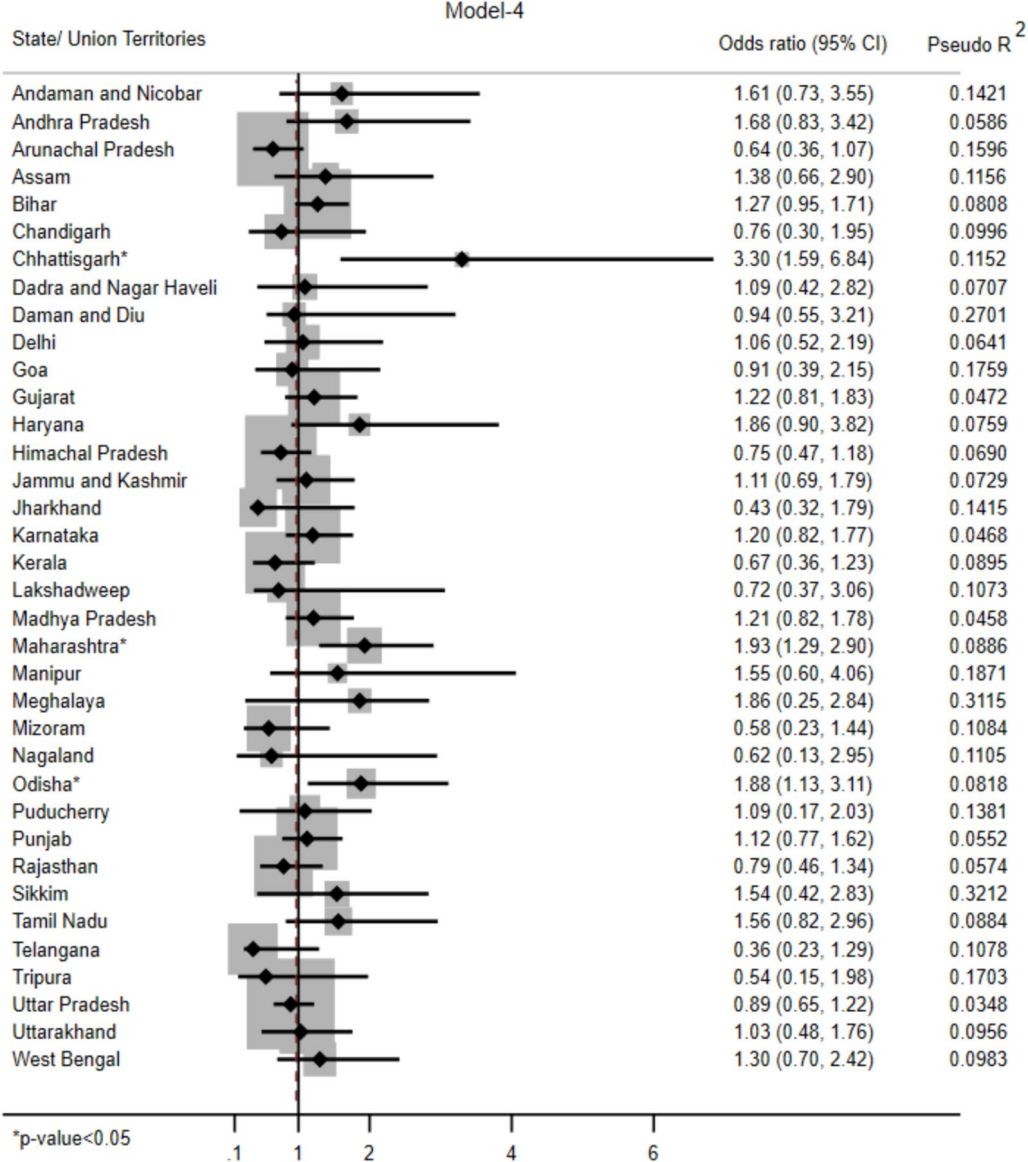
State/ Union Territory wise distribution of odds ratio showing association of anaemia with indoor air pollution among Indian population aged ≥ 45 years (Model-4)

Females had 1.86 times (AOR 1.86 (1.67–2.08)) higher adjusted odds of having anaemia. With increase in the educational status, the odds of having anaemia decreased. Participants residing in rural area had 1.47 times (AOR 1.47 (1.33–1.62)) higher odds of having anaemia. The odds of having anaemia was highest (AOR 1.37 (1.19–1.57)) among poorest participants. Participants having multimorbidity (AOR 1.86 (1.71–2.03)) and history of tobacco abuse (AOR 1.23 (1.12–1.35)) had significantly higher odds of having anaemia as per Model-4. (Fig. 3) Chhattisgarh (AOR 3.30 (1.59–6.84)), Maharashtra (AOR 1.93 (1.29–2.90)) and Odisha (AOR 1.88 (1.13–3.11)) had significantly higher odds of anaemia associated with IAP exposure. (Fig. 4).

## Discussion

This study is one of the very few studies to show the association of anaemia with indoor air pollution among older Indian adult population with a significantly large nationally representative dataset. There are several key findings of the study.

The odds of having anaemia was significantly higher among participants exposed to pollution generating source (AOR 1.30 (1.18–1.43)), household indoor smoking (AOR 1.17 (1.07–1.29. The odds was significantly 1.26 times higher (AOR 1.26 (1.15–1.38)) among participants exposed to indoor air pollution, which was higher in male (1.36 (1.15–1.61)) than female (1.21 (1.08–1.35)). Similar results were describes by studies conducted by Kelly et al. and Elbarbary et al. [13, 14]. The potential cause might be due to prolonged exposure to indoor pollution which might cause oxidative stress, inflammation, decrease absorption of iron and changes in blood parameters, such as haemoglobin levels. Pollutants present in indoor air pollution might cause haemolysis. Breathing in tiny particles (PM2.5) and gaseous pollutants can make your body more inflamed and affect how your bone marrow works [16, 17], especially if you already have conditions like diabetes or obesity. Our study documented that, the participants having multimorbidity (AOR 1.86 (1.71–2.03)) had significantly higher odds of having anaemia. Most studies have looked at how these pollutants affect inflammation in the short term [18–20], but recent research suggests they could keep causing inflammation over a long time [21–23]. This could lead to a chain reaction in your body: it might make human body to produce less of a hormone called erythropoietin, make blood cells less responsive to this hormone, and increase the levels of a protein that controls iron in the body [24, 25]. All of these things combined could mean that body makes fewer red blood cells, leading to anaemia, especially in older people.

Study by Mehta et al. revealed that increase in each 10 μg/m^3^ PM 2.5 exposure, the average prevalence of anaemia increased by 1.90% (1.43- 2.36) and the average haemoglobin concentration decreased by 0.07 gm/dL (0.05–0.09) in ecological analysis at district level. Individual level analysis produced that the odds of having anaemia was 1.09 (95% CI 1.06, 1.11) time higher with increase in each 10 μg/m^3^ PM 2.5 exposure in ambient air [26].

We found that, the central states like Uttar Pradesh, Chhattisgarh, Madhya Pradesh, Bihar had both high anaemia and IAP. The high anaemia prevalence might be due to nutritional factors, like- lack of dietary diversity, less iron rich diet and excessive milk consumption; limited health care awareness and infrastructure leading to delayed screening and diagnosis; parasitic infestation; lack of literacy and local cultural belief [27–29]. The high burden of IAP might be due to higher usage of solid/ unclean fuels (timber, straw, dung, crop residues). When these fuels are burned indoors, dangerous pollutants are released, such as fine particulate matter (PM2.5), which can worsen anaemia and cause respiratory problems [30]. Most of these states have rural participants contributing to higher traditional cooking practices with lack of proper ventilation system leading to higher IAP. Beside these, being Empowered Action Group (EAG) states having increase poverty, lack of literacy, health education, poor health indicators, lack of accessibility and affordability of improved healthcare infrastructures; participants from these states had higher anaemia and IAP exposure [31].

With increase in the educational status, the odds of having anaemia decreased. This might be due to with increase in the education status, the participants became more aware about their health status, which leads to early detection, prevention and treatment of anaemia. Participants residing in rural area had 1.47 times higher odds of having anaemia. Factors which might contribute to this were higher education, awareness, improved nutrition, better maternal education and early access to healthcare infrastructure. The odds of having anaemia was highest (AOR 1.37 (1.19–1.57)) among poorest participants. This might be due to lesser awareness, education, prevention, access to healthcare inability to pay the out-of-pocket expenditure due to treatment. Participants having multimorbidity (AOR 1.86 (1.71–2.03)) and history of tobacco abuse (AOR 1.23 (1.12–1.35)) had higher odds. This might be due to shared risk factors (inflammation, poor nutrition), interaction with iron metabolism, challenging and stigma is accessing healthcare services. Similar results were documented by various studies [32, 33].

### There are certain strengths and limitations of the study

The biggest strength lies in the large nationally representative dataset which increased its generalisability. We have established the positive association between anaemia and indoor air pollution among older adults which was noble of its kind. This study not only showed the prevalence of exposure of indoor air pollution throughout the lifetime with stratified details but also unveil the curtain from the association between anaemia and indoor air pollution which was further stratified into detailed classification as per gender. Despite these there were some limitations. Due to its cross-sectional nature temporality could not be established. We were not able to calculate the degree of exposure to pollutants. We were not able to eliminate the effect of external air pollution. Due to self-reporting type of documentation of data, the actual prevalence of anaemia might be higher. Due to the self-reporting style, there were higher probability of recall bias and social desirability bias, which could not be eliminated.

There are key policy implications and recommendations emerging from the study. Owing to the limited access to clean fuel and compulsion to use biomass owing to financial hardship in the rural areas, IAP is becoming a serious public health hazard in the nation. Nonetheless, the Indian government has made a number of efforts to improve it, including 'Pradhan Mantri Ujjwala Yojana' (PMUY) [34] and the National Programme on Improved Chula (NPIC) [35]. Due to the pandemic's devastating effects and decimation of rural people's income and way of life, studies must be done in order to adequately examine and reconsider the price of liquified petroleum gas (LPG) and subsidies for the poor. More attention should be paid to educating the public about the negative impacts of using unclean biomass energy sources, indoor air pollution, and the necessity of a functional kitchen ventilation system, among other things. In unavoidable circumstances, substitutes such enhanced chullha powered by biomass with chimneys and cookstoves can be recommended, as indicated by a previous study, which may result in reduced indoor air pollution [36]. We recommend proper clinical or community-based trial to establish the temporality, causation and natural history of association of anaemia with indoor air pollution among older adults.

## Conclusion

This study establishes convincing statistical evidence regarding positive association of anaemia with indoor air pollution among older Indian adults through a nationally representative large dataset. Interestingly, the association appears to be more pronounced among men. These findings might raise awareness and assist individuals in avoiding the negative effects of using solid/unclean fuels, inadequate ventilation, and indoor smoking. Implementing measures such as upgrading residential stoves with chimneys, providing access to clean cooking fuel, and enhancing home ventilation systems can significantly reduce exposure to indoor air pollution. The study highlights the urgency of initiating programs aimed at improving the accessibility and availability of clean fuel and technologies. By doing so, the nation can make strides toward achieving Sustainable Development Goal 7: Ensuring universal access to affordable, reliable, and modern energy services. More focused policies should be implemented towards the Empowered Action Group (EAG) states. Further evidence-based research is recommended to understand detailed causation and to establish temporality.

### Supplementary Information

Supplementary Material 1.

## Data Availability

The study utilizes nationally representative LASI survey data, which is publicly accessible and can be obtained by registering at https://iipsindia.ac.in/sites/default/files/LASI_DataRequestForm_0.pdf The processed data can be provided by the authors upon request by Dr. Pritam Halder (corresponding author).

## Abbreviations

LASI: Longitudinal Aging Study in India
PM 2.5: Particulate matter 2.5
LPG: Liquefied Petroleum Gas
MPCE: Monthly per capita expenditure
IAP: Indoor air pollution
PSM: Propensity Score Matching
AOR: Adjusted odds ratio
CI: Confidence Interval
LL: Log-likelihood
LR: Likelihood Ratio
AIC: Akaike Information Criterion
BIC: Bayesian Information Criterion
μg: Microgram
EAG: Empowered Action Group
