# Supplementary material for: Association of anaemia with indoor air pollution among older Indian adult population: multilevel modelling analysis of nationally representative cross-sectional study
Source: BMC Geriatr. 2024 Jun 29;24:567. doi: 10.1186/s12877-024-05171-2 (PMC11218345; doi:10.1186/s12877-024-05171-2)
Supplement: Supplementary file 1 — Supplementary Material 1. [file 12877_2024_5171_MOESM1_ESM.docx]

**Supplementary**

**Table S1: Distribution of participants as per Variance inflation factor**

| **Variable** | **VIF** | **1/VIF** |
| --- | --- | --- |
| **Occupation** |  |  |
| Professional and semi-professional | 1.16 | 0.86 |
| Clerical and skilled | 1.53 | 0.65 |
| Unskilled | 1.42 | 0.70 |
| **Physical activity** |  |  |
| More than once / week | 1.21 | 0.83 |
| Once / week | 1.12 | 0.89 |
| 1-3 times /month | 1.16 | 0.86 |
| Never | 1.69 | 0.59 |
| **Multimorbidity** | 1.20 | 0.83 |
| **Tobacco abuse** | 1.37 | 0.73 |
| **Alcohol consumption** | 1.28 | 0.78 |
| **Self-rated health** |  |  |
| Very good | 4.8 | 0.21 |
| Good | 6.8 | 0.15 |
| Fair | 6.04 | 0.17 |
| Poor | 3.41 | 0.29 |
| **Mean VIF** | 3.18 |  |

| **Variable** | **VIF** | **1/VIF** |
| --- | --- | --- |
| **Indoor air pollution** | 1.33 | 0.75 |
| **Age** | 1.27 | 0.79 |
| **Gender** | 1.73 | 0.58 |
| **Education** |  |  |
| Less than primary | 6.52 | 0.15 |
| Primary completed | 3.11 | 0.32 |
| Middle completed | 3.29 | 0.30 |
| Secondary school | 2.68 | 0.37 |
| Higher secondary | 2.48 | 0.40 |
| Diploma/ Graduate | 1.72 | 0.58 |
| **Residence** | 1.3 | 0.77 |
| **MPCE quintile** |  |  |
| Poorer | 1.64 | 0.61 |
| Middle | 1.67 | 0.60 |
| Richer | 1.71 | 0.59 |
| Richest | 1.81 | 0.55 |
| **Marital status** |  |  |
| Married/ in live -in | 15.36 | 0.07 |
| Widow/ separated/ divorced | 15.61 | 0.06 |
| **Health insurance** | 1.05 | 0.95 |

**Table S2: State/ Union territory wise distribution of anaemia and indoor air pollution (IAP)**

| **State/ Union Territory** | **Anaemia** | **IAP** |
| --- | --- | --- |
| Andaman and Nicobar Islands | 3.10 | 51.00 |
| Andhra Pradesh | 2.24 | 32.01 |
| Arunachal Pradesh | 2.46 | 52.14 |
| Assam | 2.00 | 53.92 |
| Bihar | 8.60 | 65.70 |
| Chandigarh | 3.06 | 25.26 |
| Chhattisgarh | 5.56 | 73.31 |
| Dadra and Nagar Haveli | 3.31 | 53.00 |
| Daman and Diu | 1.66 | 24.91 |
| Delhi | 4.40 | 24.86 |
| Goa | 3.75 | 16.46 |
| Gujarat | 8.75 | 48.99 |
| Haryana | 3.12 | 71.99 |
| Himachal Pradesh | 8.22 | 54.75 |
| Jammu and Kashmir | 5.62 | 55.86 |
| Jharkhand | 2.18 | 63.28 |
| Karnataka | 6.39 | 33.36 |
| Kerala | 2.66 | 23.66 |
| Lakshadweep | 1.10 | 57.48 |
| Madhya Pradesh | 7.33 | 68.19 |
| Maharashtra | 3.79 | 39.31 |
| Manipur | 1.74 | 45.23 |
| Meghalaya | 1.40 | 79.43 |
| Mizoram | 2.76 | 77.09 |
| Nagaland | 0.79 | 62.44 |
| Odisha | 3.01 | 55.84 |
| Puducherry | 0.12 | 17.01 |
| Punjab | 8.98 | 44.19 |
| Rajasthan | 3.53 | 63.58 |
| Sikkim | 0.56 | 29.55 |
| Tamil Nadu | 1.97 | 29.78 |
| Telangana | 0.84 | 34.04 |
| Tripura | 1.49 | 71.73 |
| Uttar Pradesh | 4.75 | 64.46 |
| Uttarakhand | 3.72 | 61.64 |
| West Bengal | 1.89 | 69.31 |
| India | 4.54 | 52.52 |
